# RETRACTION: Effect of Robotic Versus Laparoscopic Surgery on Postoperative Wound Infection in Patients With Cervical Cancer: A Meta‐Analysis

**DOI:** 10.1111/iwj.70355

**Published:** 2025-03-17

## Abstract

**RETRACTION:** HuangJ. , TanZ. , WuW. , WuX. , LiuL. , and LiC. , “Effect of Robotic Versus Laparoscopic Surgery on Postoperative Wound Infection in Patients With Cervical Cancer: A Meta‐Analysis,” International Wound Journal 21, no. 2 (2024): e14437, 10.1111/iwj.14437.PMC1082872937852784

The above article, published online on 18 October 2023, in Wiley Online Library (http://onlinelibrary.wiley.com/), has been retracted by agreement between the journal Editor in Chief, Professor Keith Harding; and John Wiley & Sons Ltd. Following an investigation by the publisher, all parties have concluded that this article was accepted solely on the basis of a compromised peer review process. The editors have therefore decided to retract the article. The authors disagree with the retraction.

**RETRACTION:** J. Huang , Z. Tan , W. Wu , X. Wu , L. Liu , and C. Li , “Effect of Robotic Versus Laparoscopic Surgery on Postoperative Wound Infection in Patients With Cervical Cancer: A Meta‐Analysis,” International Wound Journal 21, no. 2 (2024): e14437, 10.1111/iwj.14437.PMC1082872937852784

The above article, published online on 18 October 2023, in Wiley Online Library (http://onlinelibrary.wiley.com/), has been retracted by agreement between the journal Editor in Chief, Professor Keith Harding; and John Wiley & Sons Ltd. Following an investigation by the publisher, all parties have concluded that this article was accepted solely on the basis of a compromised peer review process. The editors have therefore decided to retract the article. The authors disagree with the retraction.

